# Can shortening the waiting time for gestational hypertension and preeclampsia during labor reduce adverse outcomes of conversion to cesarean section?: A retrospective cohort study

**DOI:** 10.1097/MD.0000000000049351

**Published:** 2026-06-19

**Authors:** Chun Liu, Lanxu Sun, Xin Zuo, Hongmei Shi, Yang Yao, Li Geng

**Affiliations:** a Department of Gynecology and Obstetrics, The First Affiliated Hospital of Kunming Medical University, Kunming, Yunnan, China; b Department of Gynecology and Obstetrics, Kunming First People’s Hospital, Kunming, Yunnan, China.

**Keywords:** duration of labor, hypertension during pregnancy, hypertensive disorders of pregnancy, preeclampsia, threshold effect of labor duration

## Abstract

This study aims to investigate the threshold effect of labor duration on adverse outcomes associated with cesarean sections in pregnant women with gestational hypertension and preeclampsia (GH-PE). This study conducted a retrospective cohort study on 1188 pregnant women who delivered at our hospital from January 1, 2022, to December 31, 2024. The effect of the threshold value of the labor duration for successful vaginal delivery among pregnant women with GH-PE was analyzed through curve fitting. And compare the adverse outcomes of emergency cesarean section before and after the threshold of labor duration. Among the 1188 samples, all were primiparous women who underwent cervical balloon dilation for the induction of labor. In pregnant women with GH-PE, an analysis of the threshold effect of cervical dilation duration from 2 to 4 cm on successful vaginal delivery shows that the shorter the time taken for cervical dilation, the higher the success rate of vaginal delivery. However, once this time exceeds 3.1 hour, the success rate of vaginal delivery basically remains stable. Meanwhile, it was also found that in pregnant women with GH-PE who underwent emergency cesarean section during delivery, when the duration for cervical dilation from 2 to 4 cm exceeded 3.1 hour, the incidence of postpartum hemorrhage (odds ratio: 5.97, 95% confidence interval: 1.38–25.78) and the volume of postpartum hemorrhage were higher compared to those with a duration of ≤ 3.1 hour. For primiparous women with GH-PE undergoing cervical balloon induction, the inflection point for successful vaginal delivery may be when the cervical dilation reaches 2 to 4 cm within 3.1 hour, and transfer to cesarean section before the inflection point might be possible to reduce the incidence of postpartum hemorrhage.

## 1. Introduction

Hypertensive disorders during pregnancy (HDP) are prevalent complications in obstetrics and rank among the primary causes of maternal and perinatal mortality.^[1]^ These disorders are characterized by elevated blood pressure during pregnancy, which can lead to severe complications such as placental abruption, fetal growth restriction, and eclampsia, thereby increasing the risk of death for both mother and fetus. The prevalence of HDP is reported to range from 5 to 12%.^[1,2]^ This category of disorders includes 4 main types: gestational hypertension, preeclampsia-eclampsia, chronic hypertension with superimposed preeclampsia, and chronic hypertension occurring during pregnancy.^[3]^ In China, the recent relaxation of the fertility policy, coupled with advancements in assisted reproductive technologies, has resulted in a significant increase in the number of older mothers, women utilizing assisted reproduction, multiple births, and obese pregnant individuals. Consequently, the annual incidence of HDP has been gradually rising.^[2]^

The primary objectives in managing HDP are to prevent the onset of severe preeclampsia and eclampsia while also aiming to reduce perinatal morbidity and mortality rates for both mothers and infants. For pregnant women with mild hypertension and no indications for cesarean section, vaginal delivery is recommended.^[4]^ In cases of gestational hypertension and preeclampsia (GH-PE), selective termination of pregnancy is advised after 37 weeks of gestation to mitigate the risk of adverse maternal outcomes.^[5]^ Consequently, most pregnant women experiencing HDP require labor induction to minimize complications affecting both mothers and infants, as well as to reduce the cesarean delivery rate. Studies have indicated that GH-PE can shorten both the first and second stages of labor.^[6]^ However, once labor commences, it becomes a critical period characterized by abrupt blood pressure spikes and heightened maternal risks, such as eclampsia or cardiovascular incidents.^[1]^ Extensive research has demonstrated that prolonged labor increases the incidence of eclampsia and adverse maternal-fetal complications.^[7,8]^ Furthermore, as the duration of labor extends, the rate of unfavorable pregnancy outcomes following cesarean delivery significantly rises.^[9,10]^

However, research on the adverse outcomes of prolonged labor leading to emergency cesarean sections in cases of GH-PE is limited. Therefore, we intend to conduct a retrospective study to assess the effects of GH-PE on labor duration among pregnant women who delivered at our hospital between January 1, 2022, and December 31, 2024. Additionally, we will explore the threshold effects of labor duration on successful vaginal delivery in pregnant women with GH-PE. We will also compare the adverse outcomes of emergency cesarean sections performed before and after the established labor duration threshold. Our objectives include reducing the incidence of adverse pregnancy outcomes, mitigating labor- and postpartum-related risks in pregnant women with GH-PE, and providing valuable insights to inform both clinical practice and basic research.

## 2. Materials and methods

### 2.1. Approval and registration of the study

We conducted a retrospective cohort study within the Obstetrics Department of our tertiary comprehensive hospital in China, which handles over 3500 deliveries annually. This research was conducted in accordance with the principles outlined in the Helsinki Declaration, as well as the legal and regulatory requirements of China. The Ethics Committee of the First People’s Hospital of Kunming granted approval for this study on May 23, 2025 (Ethics Approval Number: YLS2025-038-01). Data collection and research activities commenced on May 24, 2025. Patients were informed about the use of their biological samples for research purposes and provided written informed consent to participate in this study. The original data table has been uploaded to the Supplementary material, Supplementary Digital Content 1.

### 2.2. Inclusion and exclusion criteria

This research involved 10,598 expectant mothers who gave birth at the Obstetrics Department of Our Hospital from January 1, 2022, to December 31, 2024. Of these, 1188 participants satisfied the established inclusion and exclusion criteria and successfully underwent blood sample collection along with clinical data recording.

Inclusion criteria were: term pregnant women, singleton pregnancies, nulliparas, cephalic presentation, pregnant women choosing vaginal delivery with cervical dilation balloon induction, pregnant women with complete clinical data, and pregnant women who had signed the informed consent form for scientific research.

Exclusion criteria were: preterm pregnant women, multiple pregnancy, multipara, non-vertex presentation, elective cesarean section candidates, chronic hypertensive pregnant women and those with chronic hypertension complicated by preeclampsia, women opting for vaginal delivery without cervical balloon inductio, cesarean section as a transfer procedure for pregnant women with non-pregnancy-related hypertensive disorders‌, data unavailable, except for pregnant women who required a cesarean section due to failed induced labor or abnormal labor process‌.

### 2.3. Grouping

The participants were divided into 3 groups based on vaginal delivery outcomes:

1. Group VB-GH-PE (vaginal delivery group for GH-PE eclampsia): Primiparous women with GH-PE eclampsia who achieved successful vaginal delivery after cervical balloon induction.
2. Group N (control group): This group comprised nulliparous women without HDP who underwent cervical dilation balloon induction and successfully delivered vaginally.
3. Group CS-GH-PE (cesarean section group for GH-PE eclampsia): Primiparous women with GH-PE eclampsia who underwent cervical balloon induction and required cesarean section due to labor abnormalities or induction failure.
4. Divided into 2 groups based on the timing of the critical inflection point in labor duration relative to the transition to cesarean section: Group BIP, cesarean section before the inflection point in labor duration; Group AIP, cesarean section after the inflection point in labor duration.

### 2.4. Characteristics of demographics, anthropometry, and pregnancy outcomes

We gathered the following data from all participants: basic details: age, body mass index (BMI) prior to delivery, total pregnancies, total deliveries, height, and the birth weight of neonates; pregnancy-related complications: gestational diabetes mellitus, HDP, along with their classifications; labor-related information: administration of labor analgesia, fetal presentation, duration required for cervical dilation from 2 to 4 cm, duration for cervical dilation from 4 to 10 cm, length of the second stage of labor, and whether cesarean section was carried out as an intervening procedure; negative pregnancy outcomes: postpartum hemorrhage, amount of postpartum hemorrhage, neonatal asphyxia, transfer of neonates to the Neonatal Intensive Care Unit (NICU), application of intravenous antihypertensive therapy after surgery, incidence of fever within 4 days post-surgery, and issues with wound healing.

### 2.5. Instructions for induction of labor by cervical dilation balloon

The procedure of cervical dilation balloon placement: after the patient’s medical history was evaluated in detail, the informed consent was signed, and the cervical dilation balloon was placed under aseptic operation. The balloon catheter was carefully inserted into the inner mouth of the cervix, and the normal saline was slowly injected into the balloon, and 80 mL saline was injected into the inner and outer balloons, respectively. The cervical balloon was removed after 10 to 12 hours of placement.

Details of the cervical dilation balloon used in this study are as follows: Product label: cervical dilation balloon J-CRB-184000; Approval No.: 20152663937; Manufacturer: Cook Medical.

### 2.6. Diagnostic criteria-

1. Hypertensive disorders of pregnancy definition: This refers to a collection of conditions distinguished by the presence of pregnancy alongside high blood pressure.
2. Gestational hypertension definition: This form of hypertension arises after the 20-week mark of gestation, defined by a systolic blood pressure (SBP) of at least 140 mm Hg and/or a diastolic blood pressure (DBP) of 90 mm Hg or higher. Blood pressure typically returns to normal within 12 weeks following childbirth, and the diagnosis is verified postdelivery.
3. Preeclampsia-eclampsia definition: This condition involves hypertension (SBP ≥ 140 mm Hg and/or DBP ≥ 90 mm Hg) emerging after 20 weeks of pregnancy, accompanied by any of the following features: Proteinuria, defined as random urine protein measuring ≥ ++ (via dipstick), urine protein/creatinine ratio of ≥ 0.3, or 24-hour urine protein of ≥ 0.3 g; Organ dysfunction (even in the absence of proteinuria): Thrombocytopenia (platelet count < 100 × 10^9^/L); Liver dysfunction (serum transaminases ≥ 2 times normal levels); Renal dysfunction (serum creatinine exceeding 1.1 mg/dL or being ≥ 2 times the baseline value); Pulmonary edema; or new onset of headache or visual disturbances. Severe Features: SBP of 160 mm Hg or higher, or DBP of 110 mm Hg or more, along with progressive organ involvement.
4. Chronic hypertension complicating pregnancy definition: Chronic hypertension complicating pregnancy is characterized by a SBP of 140 mm Hg or more and/or DBP of 90 mm Hg or higher prior to 20 weeks of gestation, without significant worsening during the pregnancy; or hypertension that is diagnosed for the first time after the 20-week mark and continues beyond 12 weeks postpartum.
5. Chronic hypertension with superimposed preeclampsia definition: Chronic hypertension, identified prior to 20 weeks or continuing after childbirth, combined with new or worsening proteinuria after 20 weeks, or escalating hypertension accompanied by thrombocytopenia, organ dysfunction, or neurological issues.
6. Gestational diabetes mellitus definition: This condition is identified in expectant mothers who have not been previously diagnosed with pregestational diabetes mellitus through an oral glucose tolerance test conducted at 24 to 28 weeks or later. Diagnostic thresholds: Fasting glucose levels of ≥ 5.1 mmol/L; 1-hour post-glucose readings of ≥ 10.0 mmol/L; 2-hour post-glucose readings of ≥ 8.5 mmol/L. Diagnosis is established if any single value meets or exceeds these thresholds.
7. Postpartum hemorrhage definition: A blood loss of ≥ 500 mL within the first 24 hours following vaginal delivery or a minimum of ≥ 1000 mL after a cesarean delivery.
8. Neonatal asphyxia definition: The inability to begin spontaneous breathing at birth, which is a major contributor to neonatal mortality and cognitive impairments. This condition is diagnosed by an Apgar score of ≤ 7 at delivery.
9. Neonatal transfer to NICU criteria: Admission to the NICU must occur within the first 24 hours after birth.
10. Postoperative fever (cesarean section) definition: A body temperature reaching or exceeding 37.5°C recorded at least twice within 4 days following cesarean delivery.
11. Emergency cesarean section: (also known as “ labor-to-c-section”) refers to the delivery method where a pregnant woman who initially planned for vaginal delivery must urgently switch to cesarean due to sudden complications in either the mother or fetus during labor.
12. Failed induction: No labor onset after 18 hours of oxytocin intravenous infusion following membrane rupture.
13. Abnormal labor‌ (or ‌dystocia‌), refers to prolonged or arrested progress of labor during childbirth, increasing risks of adverse outcomes for both mother and fetus. It is categorized into first-stage, second-stage, and third-stage labor abnormalities.‌‌

The definitions presented above are sourced from ^[1]^.

### 2.7. Research content

Primary outcomes: curve fitting analysis to identify the threshold effect of labor duration for successful vaginal delivery in GH-PE pregnant women‌; divide the CS-GH-PE group into 2 subgroups (cesarean delivery before vs after the inflection point) and perform descriptive analysis of clinical characteristics and adverse pregnancy outcomes (postpartum hemorrhage, blood loss volume, neonatal asphyxia, neonatal NICU admission, postoperative intravenous antihypertensive medication use, fever within 4 days postpartum, poor wound healing). Multivariate binary logistic regression will be conducted for adverse outcomes with significant differences between groups.

Secondary outcomes: descriptive analysis and linear regression analysis of the time from 2 to 4 cm cervical dilation, time from 4 to 10 cm cervical dilation, and second-stage labor duration in the VB-GH-PE group and the N group‌.

### 2.8. Statistical methods

For the collection and analysis of clinical and biological data, Microsoft Excel was selected for data entry, while analyses were conducted using SPSS version 27.0 (International Business Machines Corporation). Quantitative data are presented as median values with interquartile ranges, and categorical variables are represented by frequencies and percentages. The nonparametric Kruskal–Wallis tests were employed to compare groups for quantitative variables. For qualitative variables, either chi-square tests or Fisher exact tests were utilized when the expected values were below 5. The nonparametric Spearman rank-order test was applied to compute correlations among the variables. A *P* value of < .05 was considered statistically significant. For indicators showing statistically significant differences between groups, multivariable binary logistic regression analysis was performed when the dependent variable was binary. In contrast, linear regression analysis was applied for continuous variables (such as labor duration). Subsequently, a threshold effect analysis was conducted using the Free Statistical Software Package version 2.1.1 to explore potential nonlinear relationships between labor duration (on the x-axis) and the success rate of vaginal deliveries (on the y-axis) through restricted cubic spline curves. To model this nonlinear relationship, knots for the labor duration variable were positioned at the 5th, 35th, 65th, and 95th percentiles.

Missing data description: ‌Through rigorous data verification, no missing values were observed in either group’s baseline characteristics or primary outcome measures. All variables achieved 100% complete recording. This data integrity provided a reliable foundation for subsequent statistical analyses, effectively mitigating potential bias risks associated with missing data handling.

## 3. Results

### 3.1. Flowchart of patient selection

**Figure 1.**
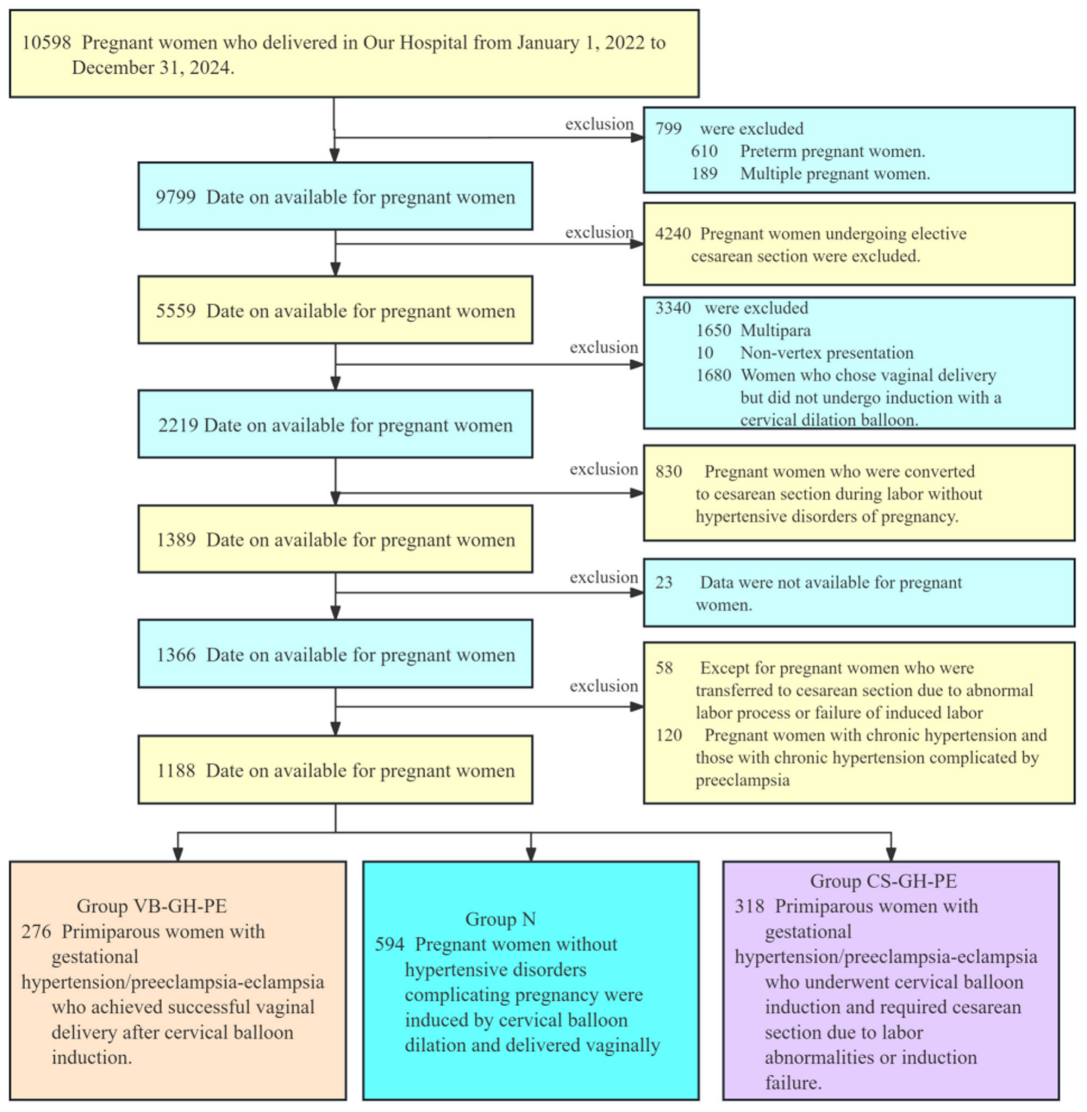
Flowchart of patient selection. GH-PE = gestational hypertension and preeclampsia.

This retrospective analysis included 10,598 expectant mothers who delivered at the Obstetrics Department of our hospital between January 1, 2022, and December 31, 2024. The exclusion criteria were as follows: premature deliveries (n = 610), multiple pregnancies (n = 189), elective cesarean sections (n = 4240), non-cephalic presentations (n = 10), multiparous women (n = 1650), women opting for vaginal delivery who did not use cervical dilation balloons for induction (n = 1680), women who were pregnant without hypertensive disorders associated with pregnancy and who underwent emergency cesarean sections during delivery (n = 830), unusable data (n = 23), cesarean sections due to fetal distress and social factors (n = 58), and pregnant women with chronic hypertension, including those with chronic hypertension complicated by preeclampsia (n = 120). Figure 1 illustrates the patient exclusion process, resulting in a total of 1188 participants in this study. Within this cohort, 276 patients were classified in the VB-GH-PE group (GH-PE pregnant women who successfully achieved vaginal deliveries using cervical dilation balloons), while the control group, referred to as the N group, consisted of 594 patients (non-hypertensive disorders of pregnancy who also achieved vaginal deliveries with the aid of cervical dilation balloons). Additionally, 318 patients were included in the CS-GH-PE group (GH-PE pregnant women who underwent cesarean sections as a secondary procedure due to abnormal labor processes or failed induction). Figure 1 is presented below.

### 3.2. Demographic and clinical characteristics of the patients

In this study, a total of 1188 patients were included in the VB-GH-PE group, CS-GH-PE group, and control group (N group), with a median age of 31 years, median prepregnancy BMI of 22.8 kg/m^2^, median height of 160 cm, median Number of pregnancies of 2, and median neonatal birth weight of 3040 g. Table 1 presents demographic and clinical characteristics of all participants, along with comparisons between the 3 groups. No statistically significant differences were observed among the 3 groups regarding maternal age, prepregnancy BMI, height, gestational diabetes mellitus, or use of labor analgesia. However, significant differences were noted in neonatal birth weight and gestational age at delivery (*P* < .05)‌.

**Table 1:** Demographic and clinical characteristics of the patients.

Maternal hypertensive disorders of pregnancy status	Total	Group VB-GH-PE	Group CS-GH-PE	Group N	*P* value
Maternal characteristics (n)	n = 1188	n = 276	n = 318	n = 594	
Median age, yrs	31.0 (28.0, 34.0)	31.0 (28.0, 35.0)	31.0 (28.0, 34.0)	31.0 (28.0, 34.0)	.635
BMI before delivery, kg/m^2^	22.8 (20.2, 26.6)	22.8 (20.3, 26.7)	23.8 (20.2, 26.6)	22.8 (20.2, 26.6)	.221
Number of pregnancies, Mean, n	2.0 (1.0, 3.0)	2.0 (1.0, 3.0)	2.0 (1.0, 3.0)	2.0 (1.0, 3.0)	.493
Maternal height, cm	160.0 (158.0, 165.0)	160.0 (158.0, 165.0)	160.0 (158.0, 165.0)	160.0 (158.0, 165.0)	.794
Gestational age at delivery, wk	37.0 (37.0, 39.0)	37.0 (37.0, 37.0)	37.0 (37.0, 37.0)	38.0 (37.0, 40.0)	< .001
Newborn birth weight, g	3040.0 (2877.5, 3190.0)	3000.0 (2850.0, 3050.0)	3000.0 (2880.0, 3100.0)	3110.0 (2930.0, 3400.0)	< .001
Gestational diabetes mellitus, %					.107
No	990 (83.3)	227 (82.2)	277 (87.1)	486 (81.8)	
Yes	198 (16.7)	49 (17.8)	41 (12.9)	108 (18.2)	
Whether to use labor analgesia, %					.758
No	747 (62.9)	170 (61.6)	205 (64.5)	372 (62.6)	
Yes	441 (37.1)	106 (38.4)	113 (35.5)	222 (37.4)	

Group VB-GH-PE (Vaginal delivery group for gestational hypertension/preeclampsia): Primiparous women with gestational hypertension/preeclampsia-eclampsia who achieved successful vaginal delivery after cervical balloon induction. Group CS-GH-PE (Cesarean section group for gestational hypertension/preeclampsia): Primiparous women with gestational hypertension/preeclampsia-eclampsia who underwent cervical balloon induction and required cesarean section due to labor abnormalities or induction failure. Group N (control group): This group comprised nulliparous women without HDP who underwent cervical dilation balloon induction and successfully delivered vaginally.

BMI = body mass index, cm = centimetre, g = gram, GH-PE = gestational hypertension and preeclampsia, HDP = hypertensive disorders during pregnancy, kg = kilogram, m = metre, n = number of patients, wk = week, yrs = years.

*P* < .05 is of significance.

### 3.3. The characteristics of the duration of labor between the VB-GH-PE group and the N group of patients

Table 2 provides a descriptive analysis comparing the labor duration across the 2 groups. Significant statistical differences (*P* < .001) were noted in the duration of cervical dilation between 2 to 4 cm, from 4 to 10 cm, as well as during the second stage of labor in pregnant women from both groups.

**Table 2:** Descriptive analysis of the duration of labor between the 2 groups.

Maternal hypertensive disorders of pregnancy status	Total	Group VB-GH-PE	Group N	*P* value
Maternal characteristics (n)	n = 870	n = 276	n = 594	
The cervical dilation time ranged from 2 to 4 cm, h	3.3 (2.4, 4.2)	2.5 (1.5, 3.4)	3.5 (3.0, 4.3)	< .001
The cervical dilation time ranged from 4 to 10 cm, h	2.5 (2.1, 3.8)	2.1 (1.3, 3.4)	3.4 (2.4, 4.2)	< .001
Time of the second stage of labor, h	0.8 (0.4, 1.3)	0.5 (0.3, 1.0)	0.9 (0.5, 1.4)	< .001

cm = centimetre, GH-PE = gestational hypertension and preeclampsia, h = hour, n = number of patients.

*P* < .05 is of significance.

### 3.4. Multivariate linear regression model of the VB-GH-PE group and the N group in terms of the duration of labor

Table 3 presents the multivariate linear regression analysis of the duration of labor between the VB-GH-PE group and the N group. Compared with the N group, the VB-GH-PE group had an average reduction of 0.98 hours in the time for cervical dilation from 2 to 4 cm, 1.38 hours in the time for cervical dilation from 4 to 10 cm, and 0.37 hours in the duration of the second stage of labor. After adjusting for age, number of pregnancies, predelivery BMI, height, gestational age at delivery, neonatal weight, gestational diabetes, and whether labor analgesia was used, the relationship between the 2 groups remained unchanged after controlling for confounding factors.

**Table 3:** Multivariate linear regression analysis of labor time between hypertensive disorder complicating pregnancy group and control group.

The total sample, n = 870 (Group VB-GH-PE, n = 276; Group N, n = 594)	Non-adjusted			Adjusted		
	*β*	*P*	95% CI	*β*	*P*	95% CI
The cervical dilation time ranged from 2 to 4 cm, h	−0.98	< .001	−0.79, −1.17	−0.98	< .001	−0.77, −1.12
The cervical dilation time ranged from 4 to 10 cm, h	−1.38	< .001	−1.15, −1.60	−1.37	< .001	−1.12, −1.63
Time of the second stage of labor, h	−0.37	< .001	−0.31, −0.44	−0.39	< .001	−0.32, −0.46

Non-adjusted: Crude model.

Adjusted: The multivariate linear regression models were modified to account for factors such as age, predelivery BMI, number of pregnancies, maternal height, newborn birth weight, Pregnancy weeks at the time of delivery, the presence of gestational diabetes mellitus, and the utilization of labor analgesia.

BMI = body mass index, CI = confidence interval, cm = centimetre, GH-PE = gestational hypertension and preeclampsia, h = hour, n = number of patients.

### 3.5. Threshold effect analysis of the effect of the duration of cervical dilation of 2 to 4 cm on the success of vaginal delivery in the GH-PE pregnant woman

Table 4 presents the threshold effect analysis of the time required for the cervix to dilate from 2 to 4 cm in the VB-GH-PE and CS-GH-PE groups, highlighting its impact on successful vaginal delivery. In the VB-GH-PE group, the number of successful vaginal deliveries among GH-PE pregnant women was n = 276. In the CS-GH-PE group, the number of cases that underwent cesarean section due to abnormal delivery or failure of induction was n = 318. The following 2 regression models indicate that when the duration of cervical dilation from 2 to 4 cm is ≤ 3.1 hours, the adjusted odds ratio (OR) for successful vaginal delivery is 0.066 (95% confidence interval [CI]: 0.029–0.153; *P* < .001), suggesting that the success rate of vaginal delivery increases as the duration of cervical dilation decreases. Conversely, when the duration of cervical dilation exceeds 3.1 hours, there is no significant association with successful vaginal delivery (OR: 0.811, 95% CI: 0.656–1.001; *P* = .053), indicating that the success rate of vaginal delivery remains unchanged as the duration of cervical dilation increases further. Therefore, it can be concluded that among pregnant women with GH-PE, a shorter duration of cervical dilation from 2 to 4 cm is associated with a higher success rate of vaginal delivery, until the duration exceeds 3.1 hours, at which point the likelihood of successful vaginal delivery becomes negligible.

**Table 4:** Threshold effect analysis of the duration of cervical dilation from 2 to 4 cm on the success of vaginal delivery in the GH-PE pregnant woman.

The cervical dilation time ranged from 2 to 4 cm, h	Adjusted model	
	OR (95% CI)	*P* value
≤3.1 h	0.066 (0.029–0.153)	< .001
>3.1 h	0.811 (0.656–1.001)	.051
Log-likelihood ratio test		< .001

Adjusted for age, BMI before delivery, number of pregnancies, maternal height, newborn birth weight, pregnancy weeks at the time of delivery,gestational diabetes mellitus, and whether to use labor analgesia.

BMI = body mass index, CI = confidence interval, cm = centimetre, GH-PE = gestational hypertension and preeclampsia, h = hour, OR = odds ratio.

**Figure 2.**
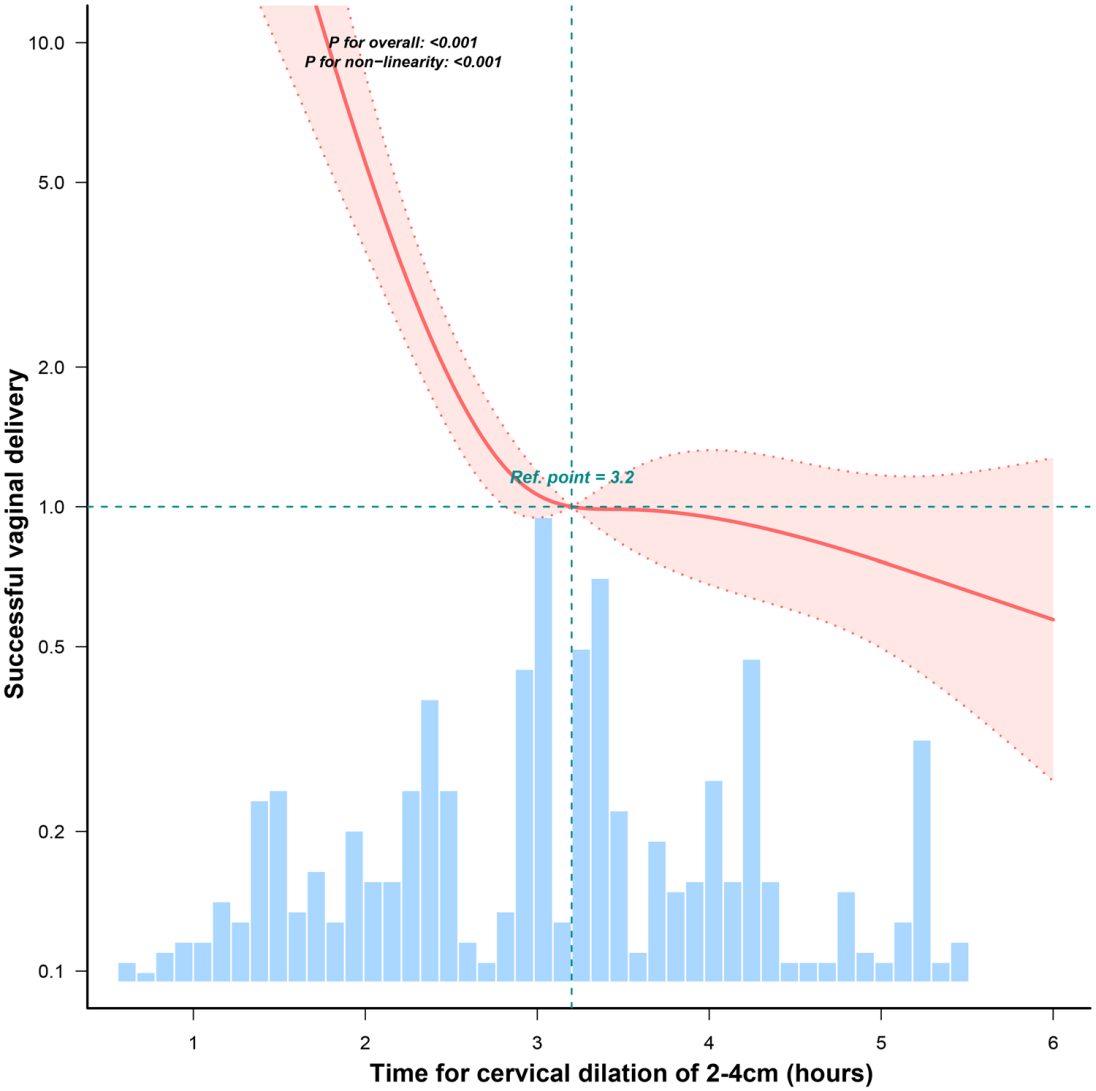
Smooth curve fitting of the relationship between the duration of cervical dilation from (2 to 4) cm and successful vaginal delivery among pregnant women with GH-PE eclampsia. The solid and dashed lines indicate the estimated values along with their respective 95% CI. Only 95% of the gathered data is represented. Adjustments for this analysis included age, predelivery BMI, maternal height, number of pregnancies, birth weight of the newborn, pregnancy weeks at the time of delivery, gestational diabetes mellitus, and the use of labor analgesia. BMI = body mass index, CI = confidence interval, GH-PE = gestational hypertension and preeclampsia.

The restricted cubic spline analysis, following multivariable adjustments, demonstrates an “L-shaped” association between the duration of cervical dilation from 2 to 4 cm and the success rate of vaginal delivery among pregnant women with GH-PE (see Fig. 2). Initially, we planned to conduct a threshold effect analysis on the duration of cervical dilation from 4 to 10 cm and the duration of the second stage of labor. However, among the total of 318 pregnant women who underwent emergency cesarean sections, only 11 experienced this during the cervical dilation period from 4 to 10 cm, and merely 2 during the second stage of labor. As a result, we did not perform the threshold effect analysis.

### 3.6. Descriptive analysis of clinical characteristics and adverse pregnancy outcomes in pregnant women of the CS-GH-PE group who underwent emergency cesarean section with cervical dilation of 2 to 4 cm lasting ≤ 3.1 hour or > 3.1 hour during delivery

In this study, a total of 318 pregnant women in the CS-GH-PE group underwent cesarean section (excluding cases where the cesarean section was performed due to abnormal labor or failure of induction, with a total of n = 58 as the indication for cesarean section). These individuals were divided into 2 categories according to the cervical dilation duration from 2 to 4 cm prior to the cesarean: BIP group included those where the dilation duration was 3.1 hour or less (n = 99), while the AIP group comprised those with a dilation duration exceeding 3.1 hour (n = 219). The average age of the participants was 31 years, their median BMI prior to delivery was recorded at 23.8 kg/m^2^, average height was 160 cm, the median number of pregnancies stood at 2, the median gestational age at delivery was 37 weeks, and the median birth weight of the newborns was 3000 g. Demographic data, clinical characteristics, and adverse pregnancy outcomes for both groups are presented in Table 5. No statistically significant differences were found between the BIP and AIP groups regarding age, predelivery BMI, height, number of pregnancies, newborn weight, pregnancy weeks at the time of delivery, gestational diabetes mellitus, or the use of labor analgesia (*P* > .05). Notably, postpartum hemorrhage and its volume exhibited statistically significant differences (*P* = .007 and *P* = .017, respectively). However, there were no significant differences observed in terms of neonatal asphyxia, transfers to the NICU, administration of postoperative intravenous antihypertensives, incidence of postoperative fever, or complications with wound healing (*P* > .05).

**Table 5:** Descriptive analysis of the general characteristics and adverse pregnancy outcomes in the emergency cesarean section group, stratified by cervical dilation duration of ≤ 3.1 h versus > 3.1 h from 2 to 4 cm in the CS-GH-PE group.

Status before and after the maternal inflection point	Total	Group BIP Cervical dilation of 2 to 4 cm time ≤ 3.1 h group	Group AIP Cervical dilation of 2 to 4 cm time > 3.1 h group	*P*
Maternal characteristics (n)	n = 318	n = 99	n = 219	
Median age, yrs	31.0 (28.0, 34.0)	31.0 (28.0, 35.0)	31.0 (28.0, 34.0)	.636
Maternal height, cm	160.0 (158.0, 165.0)	160.0 (158.0, 164.0)	160.0 (158.0, 165.0)	.914
BMI before delivery, kg/m^2^	23.8 (20.2, 26.6)	22.8 (20.3, 26.2)	23.9 (20.2, 26.6)	.530
Number of pregnancies, Mean, n	2.0 (1.0, 3.0)	2.0 (1.0, 3.0)	2.0 (1.0, 3.0)	.615
Pregnancy weeks at the time of delivery, wk	37.0 (37.0, 37.0)	37.0 (37.0, 37.0)	37.0 (37.0, 37.0)	.126
Newborn birth weight, g	3000.0 (2880.0, 3100.0)	3040.0 (2950.0, 3100.0)	3000.0 (2880.0, 3100.0)	.087
Gestational diabetes mellitus, %				.782
Yes	41 (12.9)	12 (12.1)	29 (13.2)	
No	277 (87.1)	87 (87.9)	190 (86.8)	
Whether to use labor analgesia, %				.860
Yes	134 (42.1)	41 (41.4)	93 (42.5)	
No	184 (57.9)	58 (58.6)	126 (57.5)	
Adverse pregnancy outcomes				
Postpartum hemorrhage, %				.007
Yes	26 (8.2)	2 (2)	24 (11)	
No	292 (91.8)	97 (98)	195 (89)	
Postpartum blood loss, mL	400 (400, 600)	400.0 (400, 500)	400 (400, 600)	.017
Asphyxia neonatorum, %				.177
Yes	5 (1.6)	3 (3)	2 (0.9)	
No	313 (98.4)	96 (97)	217 (99.1)	
Neonatal transfer to NICU, %	305 (95.9)	96 (97)	209 (95.4)	.761
Yes	13 (4.1)	3 (3)	10 (4.6)	
No	305 (95.9)	96 (97)	209 (95.4)	
Intravenous antihypertensive drugs were used postoperatively, %				1
Yes	3 (0.9)	1 (1)	2 (0.9)	
No	315 (99.1)	98 (99)	217 (99.1)	
Fever developed within 4 days after surgery, %				1
Yes	5 (1.6)	1 (1)	4 (1.8)	
No	313 (98.4)	98 (99)	215 (98.2)	
Poor wound healing, %				1
Yes	1 (0.3)	0 (0)	1 (0.5)	
No	317 (99.7)	99 (100)	218 (99.5)	

Group BIP: Cervical dilation of 2 to 4 cm time ≤ 3.1 h group; Group AIP: Cervical dilation of 2 to 4 cm time > 3.1 h group.

BMI = body mass index, cm = centimetre, g = gram, GH-PE = gestational hypertension and preeclampsia, h = hour, kg = kilogram, m = metre, mL = millilitre, n = number of patients, NICU = neonatal intensive care unit, wk = week, yrs = years.

*P* < .05 is of significance.

### 3.7. Analysis of a multivariate binary logistic regression model examining the association between emergency cesarean section due to cervical dilation duration exceeding 3.1 hour from 2 to 4 cm and postpartum hemorrhage in the group CS-GH-PE

Table 6 showcases the multivariate binary logistic regression model that examines the relationship between emergency cesarean sections after cervical dilation lasting more than 3.1 hour, from 2 to 4 cm, and postpartum hemorrhage within the group CS-GH-PE. Both the unadjusted and multivariate-adjusted models were utilized to confirm the reliability of the findings. Variables identified as potential confounders, informed by existing research and clinical insights, were incorporated into the analysis. Any variables exhibiting significant multicollinearity were removed. Based on the findings from tolerance and variance inflation factor tests, the final model showed no evidence of multicollinearity among the included variables. After adjusting for confounding factors, the relationship between cervical dilation duration exceeding 3.1 hour from 2 to 4 cm leading to emergency cesarean section and the incidence of postpartum hemorrhage remained unchanged (OR: 6.61, 95% CI: 1.51–28.93, *P* = .023). It suggests that in the CS-GH-PE group, a cervical dilation duration exceeding 3.1 hour from 2 to 4 cm leading to emergency cesarean section may be an independent risk factor for postpartum hemorrhage.

**Table 6:** Analysis of a multivariate binary logistic regression model for the association between emergency cesarean section due to cervical dilation duration exceeding 3.1 h from 2 to 4 cm and postpartum hemorrhage in the CS-GH-PE group.

Variable	The total sample n = 318, Cervical dilation of 2 to 4 cm time ≤ 3.1 h group, n = 99; Cervical dilation of 2 to 4 cm time > 3.1 h group, n = 219		
	Postpartum hemorrhage		
Model	*P*	OR	95% CI
Unadjusted	.017	5.97	1.38–25.78
Model 2	.013	6.48	1.48–28.29
Model 3	.013	6.49	1.49–28.33
Model 4	.012	6.61	1.51–28.93

Unadjusted: Crude model.

Model 2: Adjust for age, BMI before delivery, number of pregnancies, maternal height, pregnancy weeks at the time of delivery,newborn birth weight.

Model 3: Adjust for Model 2 + gestational diabetes mellitus.

Model 4: Adjust for Model 3 + whether to use labor analgesia.

CI = confidence interval, cm = centimetre, GH-PE = gestational hypertension and preeclampsia, h = hour, n = number of patients, OR = odds ratio.

*P* < .05 is of significance.

## 4. Discussion

This study, through a retrospective cohort analysis, thoroughly investigates the specific impact of GH-PE on the duration of the labor process and the threshold effect of labor duration on successful vaginal delivery. Compared to pregnant women without HDP, those with GH-PE experienced significantly shorter durations for cervical dilation at intervals of 2 to 4 cm, 4 to 10 cm, and during the second stage of labor. Although statistically significant differences were observed in newborn weight and gestational weeks at delivery between the 2 groups, this association remained consistent after adjusting for confounding factors using multivariate linear regression analysis. Furthermore, the results indicated that a shorter duration for cervical dilation from 2 to 4 cm was associated with a higher success rate of vaginal delivery, until a threshold of 3.1 hours was reached, beyond which the success rate of vaginal delivery diminished substantially. Finally, compared with pregnant women who underwent cesarean section after cervical dilation lasting ≤ 3.1 hours, a cervical dilation duration of > 3.1 hours may be identified as a potential risk factor for postpartum hemorrhage.

HDP refers to diseases arising from various factors, mechanisms, and pathways, with their underlying causes and processes not yet completely understood. Some researchers have suggested a “2-stage” model for the development of preeclampsia. The initial stage, known as the preclinical phase, is marked by dysfunctional remodeling of uterine spiral arteries by trophoblasts, resulting in placental ischemia and hypoxia, which subsequently prompts the release of several placental factors. The subsequent stage sees these placental factors entering the maternal bloodstream, which fosters the activation of systemic inflammatory responses and causes damage to endothelial cells, leading to the varied clinical features of PE. Presently, the primary theories concerning the causes and development of this condition include: insufficient remodeling of uterine spiral arterioles, excessive inflammatory and immune activation, and to endothelial cells.^[1]^ In contrast to individuals who undergo typical blood pressure fluctuations during pregnancy, those with preeclampsia exhibit decreased plasma renin activity, a diminished glomerular filtration rate, and heightened blood circulation with vasoconstriction, resulting in edema and hypertension.^[11,12]^ While the precise cause of preeclampsia remains unclear, abnormal attachment of the placenta and shifts in angiogenic factors during early pregnancy play a role in its development. Inadequate remodeling of the uterine spiral arteries due to remaining smooth muscle leads to diminished uteroplacental perfusion and a persistent reduction in spontaneous vasoconstrictive ability, triggering ischemia-reperfusion injury and heightened oxidative stress.^[11,13,14]^ Increased levels of circulating soluble fms-like tyrosine kinase 1, an anti-angiogenic factor produced by the placenta, result in lower levels of placental growth factor and vascular endothelial growth factor, which in turn initiates hypertension and glomerular damage.^[15]^ Due to the severe health impacts of preeclampsia on both mothers and newborns, research has focused on disease prevention measures, including lifestyle adjustments and medical management.^[11,16,17]^

In recent years, studies examining the effects of HDP on the duration of labor have been relatively scarce. A multicenter study conducted in 2012 assessed labor duration among pregnant women experiencing hypertensive disorders, revealing that the rate of cervical dilation was more rapid in those with GH-PE compared to their counterparts without such disorders.^[7]^ Furthermore, a retrospective analysis from 2002 involving 136 cases demonstrated that the average interval from labor induction to delivery was significantly reduced for women with PE, averaging 762.5 minutes as opposed to 1049.5 minutes for those without the condition,^[18]^ which is consistent with our study’s results. However, our study has achieved innovative results in analyzing the threshold effect of labor duration on successful vaginal delivery. We identified that a cervical dilation duration of 3.1 hour from 2 to 4 cm represents a critical inflection point for predicting successful vaginal delivery in pregnant women with GH-PE. Preliminary findings also suggest that converting to cesarean section before reaching this inflection point may reduce the incidence of postpartum hemorrhage. This discovery not only offers a temporal window for clinical interventions but also suggests new research directions for future studies.

Why do GH-PE lead to shortened labor duration? This phenomenon may involve multiple pathophysiological mechanisms, and the following discussion explores possible explanations from the perspectives of neuroendocrinology, vasoactive substances, and other factors. Firstly, pregnant women with preeclampsia exhibit significant sympathetic overactivity, which may directly affect uterine contraction patterns.^[19]^ Studies have shown that the resting muscle sympathetic nerve activity frequency in patients with PE (33 ± 3 bursts/min) is significantly higher than that in normal pregnant women (12 ± 2 bursts/min) and nonpregnant hypertensive patients (15 ± 3 bursts/min). This overactivation returns to normal levels rapidly after delivery. Such sympathetic overactivation may lead to stronger and more frequent uterine contractions by increasing the excitability and contraction frequency of uterine smooth muscle, thereby accelerating the progress of labor. Simultaneously, it significantly increases peripheral vascular resistance and blood pressure, potentially altering uterine-placental blood flow distribution and indirectly affecting uterine contraction characteristics.^[19]^ Secondly, abnormal expression of multiple vasoactive substances in patients with hypertensive disorders of pregnancy directly affects uterine contraction dynamics. Women who are pregnant and have preeclampsia exhibit markedly elevated concentrations of asymmetric dimethylarginine, symmetric dimethylarginine, and L-arginine in their plasma at the time of delivery when contrasted with those who are pregnant normally.^[20]^ These substances act as inhibitors of nitric oxide synthase, reducing nitric oxide production. Nitric oxide is a uterine smooth muscle relaxant, and its reduction leads to enhanced uterine contraction activity.^[20]^ Additionally, endothelial damage results in increased release of vasoconstrictors such as endothelin-1, which not only elevates blood pressure but may also directly stimulate uterine smooth muscle contraction, leading to stronger uterine contractions and accelerated labor. Thirdly, the impact of HDP on labor duration may be the result of multiple interacting factors. Due to concerns about disease progression, clinicians may more actively manage the labor process of patients with preeclampsia, such as by increasing the use of oxytocin or choosing other interventions.^[20,21]^ Furthermore, pregnant women with preeclampsia exhibit higher levels of inflammatory markers (e.g., TNF-R1, GDF-15),^[20]^ which may also directly or indirectly affect uterine contractility. In summary, shortened labor duration in pregnant women with GH-PE may be the result of multiple mechanisms acting together, involving sympathetic overactivity, imbalance of vasoactive substances, and other factors. However, the specific causes still require further research and exploration.

The highlights of this study are as follows: This research is the first to analyze the threshold effect of cervical dilation duration from 2 to 4 cm on successful vaginal delivery in pregnant women with GH-PE, providing an important temporal reference for clinical interventions. Additionally, the study found that converting to cesarean section prior to reaching the inflection point significantly reduced both the incidence and volume of postpartum hemorrhage, a finding of considerable clinical importance. This not only informs physicians’ decisions regarding labor management but also suggests new research directions for future studies.

Nonetheless, this research also has several limitations. Firstly, as a single-center retrospective cohort study, it cannot establish potential causal relationships. Secondly, although we collected data directly from the hospital’s electronic medical record system, which can be traced back to the original records and verified by 2 individuals as part of quality control measures to ensure the data’s authenticity and reliability, there are still shortcomings regarding sample diversity and the generalizability of the findings. Furthermore, the limited sample size of pregnant women with GH-PE who underwent cesarean delivery during the cervical dilation stages of 4 to 10 cm and the second stage of labor restricted our ability to assess the threshold effect related to the active phase of the first stage and the duration of the second stage of labor on achieving successful vaginal delivery.

In upcoming research, we plan to carry out broader and more in-depth investigations across several hospitals within our area to enhance the generalizability, size of the sample, and variety of outcomes. Furthermore, we intend to lengthen the follow-up duration to assess the long-term impacts on both neonatal and maternal results. Finally, we aim to incorporate pregnant women from different regions and countries to improve external validation and verify the consistency and overall applicability of our results.

In summary, this study, through an in-depth retrospective cohort analysis, has further enhanced our understanding of the impact of GH-PE on the duration of labor. the study also found that cesarean section before reaching the inflection point significantly reduced the incidence and volume of postpartum hemorrhage in pregnant women with GH-PE. This discovery holds significant clinical importance, aiding doctors in performing timely cesarean sections when necessary to reduce the risk of postpartum hemorrhage. In conclusion, the significance of this study and its assistance to peers lie in deepening our understanding of the impact of HDP, providing guidance for clinical decision-making, optimizing labor management, reducing the risk of postpartum hemorrhage, and offering directions for future research. These findings not only contribute to enhancing the safety and success rate of the childbirth process but also promote the comprehensive development of maternal and child health.

## 5. Conclusions

In conclusion, this retrospective cohort study found that among nulliparous women undergoing labor induction with cervical balloon dilation, GH-PE may shorten the duration of cervical dilation from 2 to 4 cm, 4 to 10 cm, and the second stage of labor.. Furthermore, the study identified that in pregnant women with GH-PE, a cervical dilation duration of 3.1 hours within the 2 to 4 cm range may represent an inflection point for successful vaginal delivery. Performing a cesarean section before reaching this threshold may reduce the incidence of postpartum hemorrhage. This finding provides a quantitative basis for refined labor management in pregnant women with GH-PE, assisting clinicians in more precisely determining the timing of interventions to balance the success rates of vaginal delivery against the risks of peripartum complications.

## Acknowledgments

We extend our thanks to the patients and their families for placing their trust in our research. Furthermore, we wish to recognize the valuable oversight provided by the Ethics Department and the Science and Education Department of Kunming First People’s Hospital regarding this study. Our appreciation also goes to Fengting Mu, a Master of Statistics, for her assistance with the statistical analyses presented in this paper. We are particularly grateful for the support received from the Data Collection Center of the Clinical Laboratory at Kunming First People’s Hospital in gathering the data.

## Author contributions

**Conceptualization:** Chun Liu, Lanxu Sun, Yang Yao, Li Geng.

**Data curation:** Chun Liu, Lanxu Sun, Xin Zuo, Yang Yao, Li Geng.

**Formal analysis:** Chun Liu, Hongmei Shi, Yang Yao, Li Geng.

**Funding acquisition:** Hongmei Shi.

**Investigation:** Chun Liu, Li Geng.

**Methodology:** Chun Liu, Lanxu Sun, Hongmei Shi, Yang Yao, Li Geng.

**Supervision:** Xin Zuo.

**Validation:** Chun Liu, Xin Zuo.

**Writing – original draft:** Chun Liu.

**Writing – review & editing:** Chun Liu, Lanxu Sun, Xin Zuo, Yang Yao, Li Geng.


